# Auxin Homeostasis in Arabidopsis Ovules Is Anther-Dependent at Maturation and Changes Dynamically upon Fertilization

**DOI:** 10.3389/fpls.2017.01735

**Published:** 2017-10-10

**Authors:** Emma Larsson, Adam Vivian-Smith, Remko Offringa, Eva Sundberg

**Affiliations:** ^1^Institute of Biology Leiden, Plant Developmental Genetics, Leiden University, Leiden, Netherlands; ^2^Department of Plant Biology, BioCentre and Linnean Centre for Plant Biology in Uppsala, Swedish University of Agricultural Sciences (SLU), Uppsala, Sweden; ^3^Forest Genetics and Biodiversity, Norwegian Institute of Bioeconomy Research, Ås, Norway

**Keywords:** auxin, emasculation, female gametophyte, fertilization, funiculus, ovule, PIN, YUC

## Abstract

The plant hormone auxin is a vital component for plant reproduction as it regulates the development of both male and female reproductive organs, including ovules and gynoecia. Furthermore, auxin plays important roles in the development and growth of seeds and fruits. Auxin responses can be detected in ovules shortly after fertilization, and it has been suggested that this accumulation is a prerequisite for the developmental reprogramming of the ovules to seeds, and of the gynoecium to a fruit. However, the roles of auxin at the final stages of ovule development, and the sources of auxin leading to the observed responses in ovules after fertilization have remained elusive. Here we have characterized the auxin readout in Arabidopsis ovules, at the pre-anthesis, anthesis and in the immediate post-fertilization stages, using the *R2D2* auxin sensor. In addition we have mapped the expression of auxin biosynthesis and conjugation genes, as well as that of auxin transporting proteins, during the same developmental stages. These analyses reveal specific spatiotemporal patterns of the different auxin homeostasis regulators. Auxin biosynthesis genes and auxin transport proteins define a pre-patterning of vascular cell identity in the pre-anthesis funiculus. Furthermore, our data suggests that auxin efflux from the ovule is restricted in an anther-dependent manner, presumably to synchronize reproductive organ development and thereby optimizing the chances of successful fertilization. Finally, *de novo* auxin biosynthesis together with reduced auxin conjugation and transport result in an enhanced auxin readout throughout the sporophytic tissues of the ovules soon after fertilization. Together, our results suggest a sophisticated set of regulatory cascades that allow successful fertilization and the subsequent transition of the female reproductive structures into seeds and fruits.

## Introduction

Developmental switches that terminate one developmental program in favor of the onset of another, are key processes during the differentiation of a complex multicellular organism. These switches are of particular importance in sessile plants, as they allow the plants to adapt their life strategies to changes in their environment (Huijser and Schmid, 2011). One important switch that is often neglected is the developmental arrest of the mature female reproductive structures, including the ovules and the gynoecium, before anthesis and pollination, and its subsequent release by fertilization, which triggers the onset of embryo, seed and fruit development, respectively. The developmental arrest either allows a coordinated maturation of male and female reproductive structures, or guarantees unsynchronized maturation of the two types of reproductive structures to favor outcrossing. In the hermaphrodite self-fertilizing model plant *Arabidopsis thaliana*, closing the developmental gap between male and female reproductive structures ensures that pollination occurs during the female receptive period, thereby optimizing the conditions for successful fertilization (Vivian-Smith and Koltunow, 1999; Carbonell-Bejerano et al., 2010). Successful fertilization is generally a prerequisite for embryo, seed and fruit development, and without fertilization, senescence of the entire flower usually follows (Fuentes and Vivian-Smith, 2009). Thus the developmental switch at anthesis is vital for different breeding and reproductive strategies and for the survival of a plant species.

**FIGURE 1:**
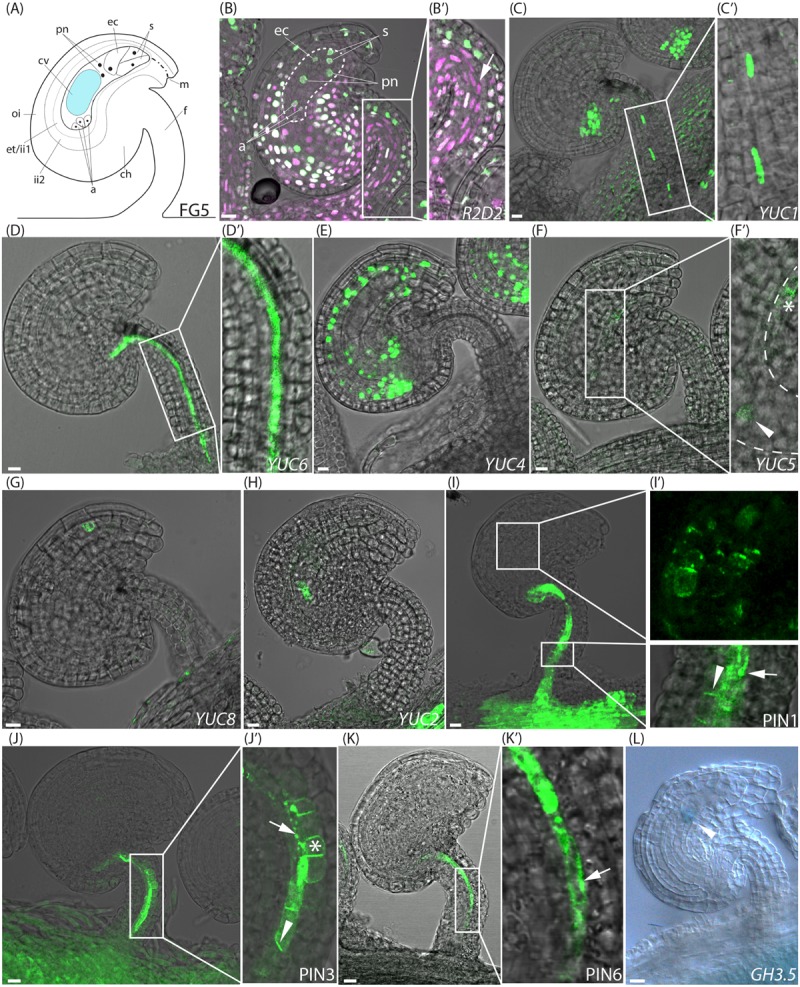
Activity of auxin related reporters at the pre-anthesis stage of FG5. **(A)** Schematic overview of an ovule at stage FG5. a, antipodal cells; ch, chalazal domain; cv, central vacuole; ec, egg cell; et, endothelium; f, funiculus; ii, inner integuments; m, micropylar end; oi, outer integuments; pn, polar nuclei; s, synergid cells. **(B–K)** Merged confocal and transmitted light images, showing the expression of different fluorescent reporter constructs in ovules carrying an embryo sac at the seven cell, eight nuclei stage. **(B′,C′,D′,F′,I′,J′,K′)** show higher magnifications of the boxed areas in **(B,C,D,F,I,J,K)**, respectively. **(B,B′)** *R2D2* activity, dotted area indicates the female gametophyte with annotations as in **(A)**, arrow indicates DII depletion in the central funicular cell files. **(C)** *YUC1pro:n3xGFP* expression in the chalazal domain of the ovule and in the third cell file of the funiculus, **(D)** *YUC6pro:eGFP* expression in the fifth cell file of the funiculus, **(E)** *YUC4pro:3xGFP* expression in the inner integuments, **(F,F′)** *YUC5pro:eGFP* expression in the micropylar end of the inner integuments (star) and in the female gametophyte (arrowhead), dashed line indicates female gametophyte, **(G)** *YUC8pro:eGFP* expression in the micropylar end of the inner integuments, **(H)** *YUC2pro:eGFP* expression in the female gametophyte, **(I,I′)** *PIN1pro:PIN1-GFP* expression around the female gametophyte and in the three most central cell files of the funiculus, arrowhead indicates basal PIN1 localization, arrow indicates internal PIN1 localization. **(J,J′)** *PIN3pro:PIN3-GFP* expression in the five most central cell files of the funiculus, arrowhead indicates basal PIN3 localization, arrow indicates internal PIN3 localization and star indicates apolar PIN3 localization. **(K,K′)** *PIN6pro:PIN6-GFP* expression in the fifth cell file of the funiculus, arrow indicates internal PIN6 localization. **(L)** DIC image of a GUS stained stage FG5 *GH3.5pro:GUS* ovule, arrowhead indicates *GH3.5* expression in the female gametophyte. Bars = 10 μm. All images are representatives of at least 10 independent samples.

In Arabidopsis, gynoecium and ovule development has been thoroughly described (Schneitz et al., 1995; Sessions, 1997). In summary, the Arabidopsis gynoecium is a bilateral hollow tube bisected into two locules by the outer replum and the inner septum. The septum harbors the placentae, from where a stalk-like structure, called the funiculus, connects the chalazal domain of the ovule to the gynoecium. The funiculus is thus the only direct route of transport for nutrients, minerals, sugars and maternal signals to the ovule and subsequently the developing embryo and seed (Khan et al., 2015). In Arabidopsis, gynoecium development starts around floral stage 5 and continues until floral stage 12, when the development is temporally arrested until fertilization (Smyth et al., 1990). The ovules are initiated from the placentae at floral stage 9, and go through a series of complex division and differentiation patterns to form the mature ovules (Schneitz et al., 1995). They consist of several different tissue types that are all collectively required for successful fertilization and subsequent embryo and seed development (Ingram, 2010). In the interior of the ovule, imbedded in several cell layers of the outer (oi) and inner integuments (ii), is the female gametophyte, which after fertilization gives rise to the embryo and the nourishing endosperm. Female gametophyte development in Arabidopsis has been divided into eight consecutive stages (FG1–FG8; Christensen et al., 1997). Stage FG5 is represented by an eight nucleate-seven-celled female gametophyte (**Figure 1A**), which is composed of one egg cell, one central cell harboring two haploid polar nuclei, two synergids and three antipodal cells. Just before anthesis, a mature diploid central cell is formed by the fusion of the two polar nuclei (Stage FG6; **Figure 3A**). At the same time an extra cell layer (ii1′) is formed between the two inner integuments, resulting in three cell layers of inner integuments, of which the inner-most cell layer (ii1) is called the endothelium (Schneitz et al., 1995). Finally at stage FG7, coinciding with anthesis, the antipodal cells degenerate, giving rise to a four-celled embryo sac surrounded by five layers of integuments (Christensen et al., 1997). Upon fertilization of the egg and central cell, respectively, the triploid central cell starts to divide without going through cytokinesis, resulting in an endosperm with free nuclei (**Figure 4A**). When the endosperm is composed of about eight free nuclei, the zygote starts to elongate and subsequently divides to develop into an embryo (Schneitz et al., 1995). Simultaneously, the gynoecium starts to grow again and develop into a fruit that will harbor, nourish, protect and finally disperse the seeds that carry the next generation (Gillaspy et al., 1993). Fruit and seed development therefore need to be tightly coordinated in order to ensure optimal success in reproduction.

As for most developmental processes in plants, dynamic responses to the phytohormone auxin have been shown to be vital for correct gynoecium, female gametophyte and ovule development (Pagnussat et al., 2009; Ceccato et al., 2013; Lituiev et al., 2013; Larsson et al., 2014; Panoli et al., 2015). Furthermore, it was recently shown that endosperm-produced auxin is important for seed coat differentiation and parthenocarpy, fruit growth without fertilization (Figueiredo et al., 2016). In accordance, several reports have shown that exogenous auxin application as well as ectopic auxin production or auxin signaling in the ovule stimulate endosperm proliferation, seed coat differentiation and parthenocarpy (Rotino et al., 1997; Vivian-Smith and Koltunow, 1999; Figueiredo et al., 2016).

Auxin responses are mediated by a family of auxin response factors (ARFs), binding to specific auxin responsive elements (AuxREs) in the promoters of their target genes (Ulmasov et al., 1997; reviewed by Roosjen et al., 2017). At low auxin concentrations, responses are blocked by Aux/IAA repressor proteins, which form dimers with activating ARFs, thus preventing them from regulating their target genes. When auxin levels rise, the phytohormone facilitates binding between the DII domain of the Aux/IAA proteins and TIR1/AFB F-box proteins that are part of an E3 ubiquitin ligase complex. This results in targeting of the Aux/IAAs for degradation by the 26S proteasome, thereby releasing the ARFs from repression (reviewed by Roosjen et al., 2017). Based on the canonical AuxRE sequence, the synthetic auxin response promoter *DR5* was produced, consisting of a 7x repeat of the element placed upstream of a *35S* minimal promoter (Ulmasov et al., 1997).

Both the *DR5rev::GFP* and *DR5v2*::Venus reporters are strongly up-regulated in Arabidopsis ovules shortly after fertilization (Dorcey et al., 2009; Fuentes and Vivian-Smith, 2009; Figueiredo et al., 2016). Together with the above mentioned data this was used to suggest a model where fertilization triggers increased auxin levels in ovules, which in turn activates phytohormone signaling as well as cell division and growth in both ovules and fruit (Fuentes and Vivian-Smith, 2009; Figueiredo et al., 2016). However, since the *DR5* promoter depends on the presence of different ARFs, which dynamically changes in ovules around fertilization (Goetz et al., 2006), and on the *35S* minimal promoter, which appears inactive within the female gametophyte (Roszak and Köhler, 2011), the analyses of *DR5* expression might not provide a comprehensive assessment of all the auxin signaling events occurring in the ovule during the pre-anthesis and post-fertilization stages.

More recently, the new ARF-independent ratio-metric *R2D2* auxin sensor system was developed (Liao et al., 2015). The system is based on one single construct in which the promoter of the cell division-correlated *RPS5A* gene drives both the expression of the auxin sensitive Aux/IAA DII domain (Brunoud et al., 2012) linked to a nuclear targeted fluorophore (DII:n3x-Venus; green) and an auxin-resistant version of the identical domain linked to another nuclear targeted fluorophore (mDII:ntdTomato; magenta). Cells with low auxin will thus accumulate relatively equal amounts of both fusion proteins (green and magenta, respectively), while cells with high auxin levels only will accumulate the auxin-resistant fusion protein (magenta). Auxin-mediated readout can thus be measured as the ratio between the green and magenta fluorophore protein emissions.

Although auxin responses have been analyzed in ovules before and after fertilization, reports of auxin action at narrow time points around fertilization are still missing and knowledge about auxin biosynthesis, transport and the responses to auxin around fertilization is rather scarce. Thus, to understand the roles of auxin during the transition from ovule and gynoecium development into seed and fruit growth, we monitored the auxin-mediated readout using the *R2D2* sensor, and the expression of key regulators of auxin biosynthesis, transport and conjugation in ovules before and during the developmental arrest of ovules and gynoecia, as well as immediately after the re-initiation of growth and development of the ovules into seeds and the gynoecium into a fruit. Our results show low auxin sensor detection in pre-anthesis ovules, except for in the funiculus, where the auxin readout together with different modules of auxin biosynthesis and transport mark the different precursor tissues of the vascular system. At anthesis, anther-dependent signals repress auxin export from the ovule through the immature phloem in the funiculus, presumably to fine tune growth and development of ovules and gynoecia to ensure successful coordination of the self-fertilization process. After fertilization, auxin available for sensing accumulates inside the ovule, which stimulates rapid potentiation of the auxin responses to coordinate embryo, seed and fruit development.

## Materials and Methods

### Plant Material and Growth Conditions

*Arabidopsis thaliana* ecotype Columbia (Col) was used throughout this study unless otherwise stated. The *R2D2* (Liao et al., 2015), *YUC1pro:n3xGFP*, *YUC2pro:eGFP-GUS*, *YUC4pro:n3xGFP*, *YUC5pro:eGFP-GUS*, *YUC6pro:eGFP-GUS*, *YUC8pro:eGFP-GUS*, *YUC10pro:n3xGFP*, and *YUC11pro:eGFP-GUS* (Robert et al., 2013), *PIN1_pro_*:*PIN1-GFP* (Benková et al., 2003), *PIN3pro*:*PIN3-GFP* (Zádníková et al., 2010), *PIN6pro*:*PIN6-GFP (*Sawchuk et al., 2013*), GH3.5pro:GUS* (Gutierrez et al., 2012), *SUC2pro:GFP* (Imlau et al., 1999), *cer6-2* (Preuss et al., 1993), and *FIS2pro:FIS2-GFP* (Portereiko et al., 2006) lines have been described before. For clarity, all reporter constructs have been depicted in Supplementary Figure 1. Of these lines, *cer6-2* is in the Landsberg *erecta* (L.*er*) ecotype (Preuss et al., 1993). Plants were germinated on petri dishes with 0.5 × Murashige and Skoog (MS) medium supplemented with 1% sucrose and 0.8% agar for 1–2 weeks. Seedlings were then transplanted to soil and grown under controlled long-day growth conditions (16/8 h photoperiod at 110 μmol m^-2^ s^-1^ and 22°C). Samples were collected from the primary inflorescence around 4 weeks after transplantation to soil.

### Sample Preparations

Ovules were hand dissected from gynoecia or fruits and mounted in water (for confocal microscopy), or in 30% glycerol (for histochemical analysis). For analysis of PIN localization and *R2D2* signaling, dissected gynoecia and fruits were fixed in buffered paraformaldehyde for 1 h as described in Larsson et al. (2014). Fixation kept the fluorescent signal stable for up to 24 h. For GUS staining, whole inflorescences were collected in ice-cold 90% acetone and stained as in Larsson et al. (2014). At least 10 samples were analyzed for each reporter at each stage.

### Microscopy and Imaging

Confocal laser-scanning micrographs of ovules were obtained with a Zeiss 780 Inverted Axio Observer with supersensitive GaASp detector. For GFP, Venus and TdTomato detection, 488, 514, and 561 nm argon lasers were used for each respective excitation, and emissions were detected between 493 and 598 nm for GFP, between 526 and 544 nm for Venus and between 570 and 632 nm for TdTomato. Using a C-Apochromat 40× water immersion objective (numerical aperture = 1.2) confocal scans were performed with the pinhole at 1 Airy unit. Presented images show either a single focal plane or a 3D reconstruction of individual images taken as a z-series and processed using the ZEN2011 software.

Activity of the *SUC2pro:GFP* reporter in FG5 stage ovules was analyzed using a Leica DMI4000 inverted microscope (HXC PL fluotar 10× objective) with differential interference contrast (DIC; Nomarski) optics, a Leica DFC360FX camera, and LAS AF (Leica Microsystems) software. GFP was detected using an L5 fluorescein isothiocyanate/GFP band-pass filter.

GUS staining was analyzed using a Zeiss Axioplan microscope with DIC (Nomarski) optics, a Leica DFC295 camera and LAS core imaging software.

Adobe Illustrator CS6 and Adobe Photoshop CS6 were used to assemble photographs and to add arrows to indicate details.

## Results

### Sites of *YUC*-Mediated Auxin Biosynthesis in Pre-anthesis Ovules Is Only Partially Reflected by Auxin Readout

To study the auxin dynamics in ovules before and after fertilization, we first analyzed the expression of the *R2D2* reporter at consecutive stages pre- and post-fertilization. At stage FG5 (**Figure 1A**), when the gynoecium development is arrested, both the DII-Venus and mDII-Tomato accumulate throughout most parts of the ovule (**Figures 1B,B′** and Supplementary Figure 2A). This implies that auxin accumulation in ovules is relatively limited right before anthesis. However, a reduced DII-Venus/mDII-Tomato fluorescence ratio, as a result of DII depletion, in the three central-most cell files of the funiculus (arrow in **Figure 1B′**), as well as in a few cells of the chalazal domain, and to some extent in the endothelium (**Figure 1B**), indicates elevated levels of auxin detection in these tissues. The observed DII depletion pattern at stage FG5 essentially agrees with previous reports using *DR5* reporters (Pagnussat et al., 2009; Lituiev et al., 2013).

The importance of auxin biosynthesis for the observed DII depletion sites in FG5 stage ovules was assessed by analyzing the expression of the *YUCCA* (*YUC*) genes. These encode enzymes that catalyze the final rate-limiting step of the primary auxin biosynthesis pathway in Arabidopsis (Zhao, 2012), and have been implicated as key regulators of both floral organ and embryo development (Robert et al., 2015). Expression patterns of three nuclear targeted *n3xGFP* (*YUC1, 4*, and *10*) and eight cytosolic (*YUC2,3,5-9*, and *11*) *eGFP-GUS* reporters (Robert et al., 2013) were analyzed in ovules at consecutive stages around fertilization. In FG5 stage ovules, two of the auxin biosynthesis reporters, *YUC1pro:n3xGFP* and *YUC6pro:eGFP-GUS*, are active in central tissues of the funiculus (**Figures 1C,C′,D,D′**), suggesting that YUC1 and YUC6 may contribute to the auxin readout detected by the *R2D2* sensor system in these tissues. Interestingly, the expression of both reporters is confined to single, but complementary cell files. For simplicity, we have numbered the approximate positions of these cell files from 1 to 7, starting from the adaxial side of the funiculus (Supplementary Figure 3A). The *YUC1* reporter is according to this numbering scheme expressed within the cells of file 3 while the *YUC6* reporter is expressed within the cells of file 5 (compare **Figure 1C′** with **Figure 1D′**). Additionally, activity of other *YUC* genes can be detected also outside the *R2D2* sensing domain. *YUC4pro:n3xGFP* shows expression throughout the inner integuments (**Figure 1E**) while *YUC5pro:eGFP-GUS* and *YUC8pro:eGFP-GUS* are expressed only in the micropylar end of the inner integuments (**Figures 1F,F′**, star, and **Figure 1G**). The observed *YUC8* expression is similar to previous reports of its expression during ovule development (Panoli et al., 2015). Likewise *YUC2pro:eGFP-GUS* is expressed in the female gametophyte together with *YUC5* (**Figures 1F,F′**, arrowhead, and **Figure 1H**; Pagnussat et al., 2009; Panoli et al., 2015). The eGFP signal of these two reporters appears to be localized in the chalazal end of the female gametophyte, which most likely represents the central cell. As the eGFP produced by these reporters is targeted to the cytosol, and the cytosol of the large central cell is pressed to the plasma membrane by the central vacuole, it is not surprising that the eGFP signal is restricted to a subpart of this cell. Nevertheless, despite the *YUC* activity in the female gametophyte and the inner integuments, no specific auxin-mediated DII depletion could be detected in these tissues using *R2D2*.

### Funicular Auxin Export and Auxin Conjugation in the Female Gametophyte Moderate Auxin Readout in Pre-anthesis Ovules

To analyze if the spatial discrepancies between auxin biosynthesis and the auxin-mediated DII depletion detected in pre-anthesis ovules may be facilitated by polar auxin transport (PAT) away from the sites of synthesis, we assessed the expression and subcellular localization of members of the PINFORMED (PIN) family of auxin efflux carriers (Friml et al., 2003). These are continuously recycled between the plasma membrane and endosomes, thus enabling a flexibility of the transport system to be dynamic in both activity and direction (Michniewicz et al., 2007; Kleine-Vehn et al., 2009). We selected the six members of the PIN-family, PIN1-4 and 6-7, that have been shown to be crucial for the polar cell-to-cell auxin transport via their polar plasma membrane localization (Simon et al., 2016), and analyzed their localization using available translational fusion reporters. As previously reported (Ceccato et al., 2013), both *PIN1pro:PIN1-GFP* (Benková et al., 2003) and *PIN3pro:PIN3-GFP* (Zádníková et al., 2010) expression can be detected in the ovule before anthesis (**Figures 1I,J**). PIN1 is strongly expressed in the chalazal domain, and only weakly in a few cells of the inner integuments surrounding the chalazal part of the female gametophyte (**Figure 1I′**). PIN3 expression is absent from the actual ovule. However, both PIN1 and PIN3 are strongly expressed in the central cell files of the funiculus, where they localize in a polar orientation at the basal plasma membrane in at least one cell file, presumably canalizing auxin transport toward the placenta (**Figures 1I′,J′**, arrowheads). As both PIN1 and PIN3 are also localized to intracellular structures in some cell files (**Figures 1I′,J′**, arrows), the number of PIN expressing cells showing basal, apolar (**Figure 1J′**, star) or intracellular PIN localization was counted (Supplementary Figure 3). Strikingly, PIN1 is basally localized in a majority of the PIN1 expressing cells within file 3 (66%), while PIN3 expression is absent from these cells. In contrast, PIN3 is basally localized in the neighboring cells of the central-most cell file (file 4), while PIN1 is mainly internalized in these cells (68%). PIN1 is absent from files 2 and 6, while PIN3 is basally localized in a majority of the PIN3 expressing cells in file 2 (74%), and apolarly localized in most of the PIN3 expressing cells in file 6 (92%). This may suggest that auxin is exported from those cells in an undirected manner. Furthermore, both PIN1 and PIN3 are mainly internalized in cell file 5 (83 and 81%, respectively; **Figures 1I′,J′**, arrows), suggesting that cells in this file are not important for auxin flow through the funiculus before anthesis. *PIN6pro:PIN6-GFP* (Sawchuk et al., 2013) is also expressed in the funiculus, but only in cells of file 5, where it is localized internally, presumably at the ER membrane (**Figures 1K,K′**). No expression of PIN2, PIN4 or PIN7 could, however, be detected in ovules at any of the stages analyzed. Significantly, the polar localization of PIN1 and PIN3 within three cell file locations of the funiculus strongly supports the hypothesis that at least some auxin produced in the ovule before anthesis is transported and canalized through specific cell files in the funiculus.

As conjugated auxin has been detected by immunolocalization in developing ovules (Aloni et al., 2006), available transcriptional reporters of genes encoding family members of the auxin-conjugating enzymes GRETCHEN HAGEN3 (GH3) were analyzed to test if the auxin produced in the pre-anthesis integuments or in the female gametophyte may be conjugated and stored inside the ovules as inactive conjugates. Interestingly, *GH3.5pro:GUS* (Gutierrez et al., 2012) is specifically expressed in the female gametophyte before anthesis (**Figure 1L**), suggesting that auxin produced in this tissue, is compartmentalized as conjugates to maintain low levels of active auxin here, and potentially to store inactive auxin for later time points.

### Different Sets of Auxin Biosynthesis Genes and Auxin Transporters Mark Different Vascular Precursor Cells in the Pre-anthesis Funiculus

**FIGURE 2:**
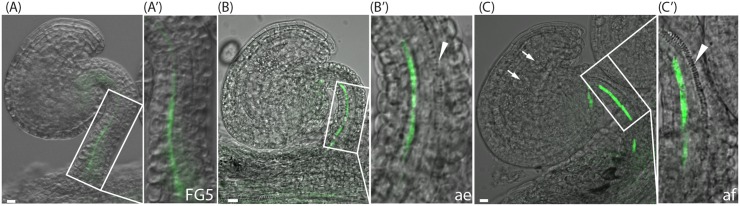
*SUC2pro:GFP* expression and xylem differentiation in ovules at the pre-anthesis stage, after emasculation and post-fertilization. **(A–C)** Merged confocal and transmitted light images of *SUC2pro:GFP* ovules at pre-anthesis, stage FG5 **(A,A′)**, at stage FG7after emasculation (ae) **(B,B′)**, and at two-nucleate endosperm stage, shortly after fertilization (af) **(C,C′)**. **(A′–C′)** show higher magnifications of the boxed areas in **(A–C)**. Green indicates *SUC2* expression in the third cell file, arrowheads indicate differentiating xylem in the fifth cell file, arrows in **(C)** indicate endosperm nuclei. Bars = 10 μm. All images are representatives of at least 10 independent samples.

Interestingly, just as the *YUC* genes, the different PIN proteins are expressed in different cell files of the funiculus. Furthermore, the different PIN proteins show different cellular localization in these files. Despite the importance of the funiculus as the sole connection between the ovule and the mother plant, little is known about the tissues of the funiculus, and particularly the order of the functionally different cell types of the vascular system running through the funiculus. Hence, to determine the identities of the cell files expressing the different reporters, we analyzed specific characteristics of xylem and phloem tissues. The water and mineral transporting xylem tracheary elements (TEs) show a distinct pattern of secondary cell wall deposition that can be visualized through normal light microscopy. In the Arabidopsis funiculus, one single TE file is gradually differentiating in the center of the funiculus. It starts in the chalazal part of the ovule at anthesis, extends through the funiculus, and is completed only after fertilization (Fuentes and Vivian-Smith, 2009; **Figures 2A–C**, arrowhead). To gain positional information about the phloem in relation to xylem, we used the *SUC2pro:GFP* reporter which is expressed in phloem companion cells of the funiculus (Imlau et al., 1999; Werner et al., 2011). The GFP protein produced by this reporter has been shown to translocate from the companion cells into the neighboring sieve elements and subsequently partition between different sink tissues (Imlau et al., 1999). We found that expression of *SUC2pro:GFP* can be detected in one single cell file from stage FG5 to after fertilization (**Figures 2A–C**), which is in accordance with Werner et al. (2011). However, before anthesis the signal is weak and mainly restricted to the basal part of the funiculus (**Figures 2A,B**), whereas the signal becomes stronger shortly after fertilization, and subsequently extends into the chalazal part of the ovule (**Figure 2C**). Interestingly, the cells marked by *SUC2pro:GFP* activity belong to cells of file 3, which are also expressing *YUC1* (**Figures 1C,C′**) and basally localized PIN1 proteins (**Figure 1I′** and Supplementary Figure 3B), while PIN3 is absent from this cell file (**Figure 1J′** and Supplementary Figure 3C). The xylem TE precursor cells, on the other hand, are localized to cells in file 5 (**Figures 2B,C** and Supplementary Figure 3A), express *YUC6* (**Figures 1D,D′**) and have highly internalized PIN1, PIN3, and PIN6 proteins at stage FG5 (**Figures 1I–K′** and Supplementary Figures 3B,D). The xylem and the presumptive phloem strand in the funiculus are separated by a single cell file that lacks the characteristics of either xylem or phloem, and most likely retains a cambial fate. These cells bear internally localized PIN1 and basally localized PIN3 (Supplementary Figures 3B,C). Together this suggests that different modules of the auxin biosynthesis pathway, and polar auxin transport network, are associated with the specification of vascular precursor cells that have different cell fates within the Arabidopsis funiculus.

### Anthers Stimulate PIN Internalization in the Funiculus at Anthesis

**FIGURE 3:**
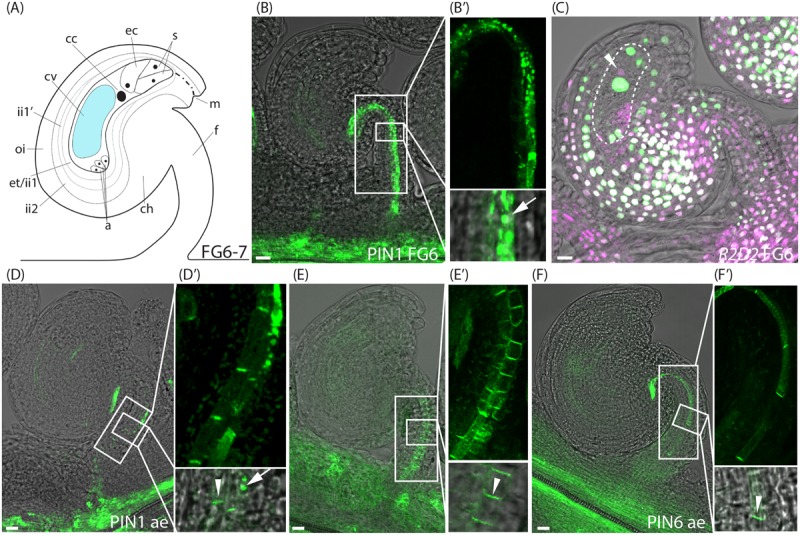
Auxin sensing and transport in ovules at the pre-anthesis stage and after emasculation. **(A)** Schematic overview of an ovule at stage FG6-7. a, antipodal cells; cc, central cell; ch, chalazal domain; cv, central vacuole; ec, egg cell; et, endothelium; f, funiculus; ii, inner integuments; m, micropylar end; oi, outer integuments; s, synergid cells. **(B,C)** Merged confocal and transmitted light images of stage FG6 *PIN1pro:PIN1-GFP* **(B,B′)** and *R2D2* **(C)** ovules just before anthesis. **(B′)** Shows higher magnifications of the boxed areas in **(B)**, arrow indicates internal PIN1 localization. Dotted area in **(C)** indicates the female gametophyte, arrowhead in **(C)** indicates DII depletion in the egg cell. **(D–F)** Merged confocal and transmitted light images of *PIN1pro:PIN1-GFP* **(D,D′)**, *PIN3pro:PIN3-GFP* **(E,E′)**, *PIN6pro:PIN6-GFP* **(F,F′)** ovules after emasculation. **(D′–F′)** show higher magnifications of the boxed areas in **(D–F)**, respectively. Arrowheads indicate basal PIN1, PIN3, and PIN6 localization, respectively, arrow indicates internal PIN1 localization. Bars = 10 μm. All images are representatives of at least 10 independent samples.

Even though PIN1 has a predominantly polar localization in the phloem cells (file 3) of the funiculus at stage FG5, it is also highly internalized in files 4 and 5 (**Figure 1I′** and Supplementary Figure 3B). As the flower approaches anthesis and the diploid central cell is formed through the fusion of the two polar nuclei (stage FG6, **Figure 3A**), PIN1 becomes increasingly internalized in all three cell files of the funiculus where it is expressed (**Figures 3B,B′**), while the expression of the *YUC* genes remains unchanged (Supplementary Figure 4). We also detected at this stage that the egg cell shows DII depletion (arrowhead, **Figure 3C** and Supplementary Figure 2B), which is in contrast to the rest of the nuclei within the female gametophyte (**Figure 3C**). Except for the decreased DII signal in the egg cell, the *R2D2* sensor expression is indistinguishable at stage FG6 from that of stage FG5. These results essentially agree with previous studies of auxin responses in unfertilized ovules using the *DR5rev* reporters (Dorcey et al., 2009; Fuentes and Vivian-Smith, 2009; Figueiredo et al., 2016). To our knowledge, however, an auxin accumulation and/or auxin-mediated activity toward the DII domain of the AUX/IAA proteins in the unfertilized egg cell have not been reported before.

To assess PIN localization and *R2D2* accumulation in mature unfertilized ovules at stage FG7, we first tested to emasculate the flowers before anthesis, and then analyzed PIN localization and *R2D2* expression 24 h later. Surprisingly, while the *R2D2* expression pattern after emasculation remains unchanged compared to FG6 (Supplementary Figure 2C), a significantly larger amount of the funicular PIN1, PIN3 and PIN6 proteins localize at the plasma membrane in a polarized manner in all cell files (**Figures 3D–F′** and Supplementary Figures 3B–D). We could not detect any differences in *YUC* expression before and after emasculation (Supplementary Figure 4).

To distinguish between putative emasculation-induced effects and developmentally activated changes in PIN localization, we crossed the PIN reporters into the conditional male sterile line *eceriferum6-2* (*cer6-2*, also known as *pop1*; Preuss et al., 1993; Vivian-Smith et al., 2001). Since *cer6-2* pollen are unable to hydrate on the stigma at low humidity, the timing of fertilization of *cer6-2* egg and central cell can be controlled by the application of wild type pollen. This line has been used extensively before to control male fertility and to study both fertilization-independent fruit growth and auxin responses around fertilization (Vivian-Smith et al., 2001; Dorcey et al., 2009). Similar to wild type, PIN1 in *cer6-2* funiculi is polarly localized in cells of file 3 at the pre-anthesis stages (Supplementary Figures 5A,A′). As the *cer6-2* flower approaches anthesis, internal PIN1 localization increases from 44 to 82% in file 3, from 68 to 94% in file 4 and from 83 to 95% in file 5 (aa; Supplementary Figures 5B,B′, 3B), which is in accordance with what we observe in wild type flowers. Also the expression of PIN3 and PIN6 is similar in pre-anthesis ovules of *cer6-2* and in wild type, and cellular localization is essentially unchanged when observed at anthesis in *cer6-2* (Supplementary Figures 5D–H′, 3C,D), except that the basal localization of PIN3 increases from 7 to 85% and from 8 to 52% in cells of files 5 and 6, respectively, at stage FG7 (Supplementary Figure 3C). Emasculation of *cer6-2* before anthesis results in a similar increase of all the PIN proteins being localized to the basal plasma membrane of the vascular precursor cells within the funiculus (Supplementary Figures 5C,C′,F,F′,I,I′), as observed in emasculated wild type funiculi. The fact that PIN1 and PIN6 are highly internalized in the *cer6-2* funiculus at stage FG7, suggests that auxin export through the funiculus and particularly through the immature phloem is retarded at anthesis, and that emasculation and not developmental timing, prevents this repression of auxin efflux.

### Auxin Readout Increases Rapidly in Ovules after Fertilization, While *YUC* Expression Remains at Steady State

**FIGURE 4:**
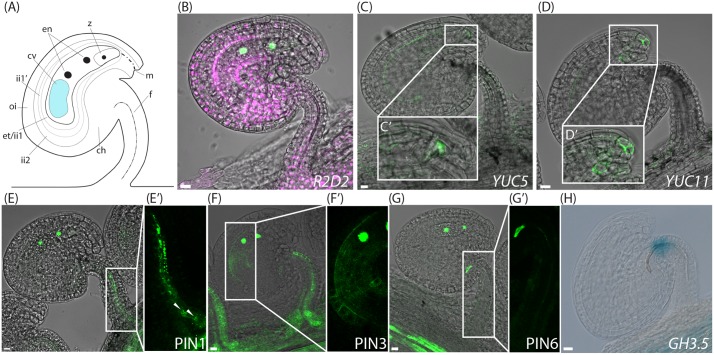
Auxin sensing, biosynthesis, transport and conjugation in ovules post-fertilization. **(A)** Schematic overview of an ovule after the first nuclear division of the central cell shortly after fertilization, ch, chalazal domain; cv, central vacuole; en, endosperm nuclei; et, endothelium; f, funiculus; ii, inner integuments; m, micropylar end; oi, outer integuments; z; zygote. **(B,G)** Merged confocal and transmitted light images of *R2D2* activity **(B)**, *YUC5pro:3xGFP* expression in the developing endosperm and in the micropylar end of the inner integument tip cells **(C,C′)**, *YUC11pro:3xGFP* expression in the micropylar end of the inner integuments **(D,D′)**, *PIN1pro:PIN1-GFP* expression in the funiculus **(E,E′)**, *PIN3pro:PIN3-GFP* expression in the funiculus and in the inner integuments **(F,F′)**, *PIN6pro:PIN6-GFP* expression **(G,G′)** in ovules at the two nucleate endosperm stage post-fertilization. **(H)** DIC image of a GUS stained *GH3.5pro:GUS* ovule at the two nucleate endosperm stage post-fertilization showing GH3.5 expression in the upper part of the funiculus. **(C′–G′)** Show higher magnifications of the boxed areas in **(C–G)**, respectively. Arrowheads in **(E′)** indicate basal PIN1 localization. Bars = 10 μm. All images are representatives of at least 10 independent samples.

Since emasculation has such a profound effect on PIN localization, we used the *cer6-2* line instead of emasculation of wild type flowers to control the timing of pollination and to analyze the spatiotemporal activity of auxin related reporters after fertilization. Shortly after fertilization, when the endosperm nucleus has divided once, but before the first zygotic cell division (**Figure 4A**), the DII accumulation is dramatically decreased in all sporophytic tissues, while it remains strong in the dividing endosperm nuclei (**Figure 4B** and Supplementary Figure 2D). This is consistent with observations of the *DR5* response reporter in sporophytic tissues after fertilization (Fuentes and Vivian-Smith, 2009; Figueiredo et al., 2016), and indicates that auxin levels may rapidly increase in these tissues soon after fertilization.

To establish if the observed fertilization-mediated tissue-specific DII depletion is based on elevated auxin biosynthesis, we analyzed the expression of the *YUC* reporters after the first endosperm nuclear division. Strikingly, fertilization does not enhance or change the expression of the majority of the analyzed auxin biosynthesis genes (Supplementary Figure 4). However, *YUC5* expression is subtly broadened compared to before fertilization. In addition to the already established expression in the embryo sac and the inner integuments, one or a few cells also express the *YUC5* reporter in the inner integument (ii1) tip cells, adjacent to the zygote (**Figures 4C,C′**). Furthermore, *YUC11*, which is not expressed before anthesis (Supplementary Figures 4W,X), becomes induced in the micropylar end of the integuments (**Figures 4D,D′**). This suggests that the rapid DII depletion in the sporophytic tissues of the ovules shortly after fertilization (**Figure 4B**) may not be initiated by *de novo* auxin biosynthesis, except in the case of the micropylar integument cells. In accordance with previous reports (Robert et al., 2013; Figueiredo et al., 2015), we did see increased *YUC10* expression in the developing endosperm (Supplementary Figure 4V). However, surprisingly, this induction occurs only after the third endosperm nuclear division, at the eight endosperm nuclei stage (compare Supplementary Figures 4U,V), and appears therefore not to contribute to the rapidly induced DII depletion shortly after fertilization.

### PIN-Mediated Auxin Transport Is Activated in the Integuments and Repressed in the Funiculus upon Fertilization

In contrast to the relatively constant levels of *YUC* expression, PIN expression is dramatically altered after fertilization (**Figure 4**). To chronicle the changes in PIN expression, we introduced the *FIS2pro:FIS2-GFP* reporter (Portereiko et al., 2006) into the *PINpro:PIN-GFP* expressing *cer6-2* plants. *FIS2* is specifically expressed in the central cell and in the free nuclei of the developing endosperm (Luo et al., 2000), and can thus specifically track the number of nuclear divisions occurring in the endosperm, and provide estimates on the time elapsed after fertilization. Already after the first endosperm nuclear division, the expression of both PIN1 and PIN6 in the funiculus decreases substantially (**Figures 4E,E′,G,G′**). Polar PIN1 localization can be detected in a few cells in the basal part of the funiculus, close to the connection to the septum (**Figure 4E′** arrowheads), whereas it is otherwise weakly expressed and internalized in 73–95% of the cells (**Figures 4E,E′** and Supplementary Figure 3B). At this same stage no PIN6 expression can be detected, except for within a few cells of the junction between the chalazal domain and the funiculus (**Figures 4G,G′**). However, some internal PIN6 localization can be visualized if the exposure time is increased substantially (Supplementary Figure 3D). In contrast, PIN3 expression remains present in the funiculus, and its localization is essentially as at anthesis (**Figure 4F** and Supplementary Figure 3C). Additionally, at this stage PIN3 also appears in the endothelium, surrounding the embryo sac (**Figures 4F,F′**), indicating that auxin transport is induced around the developing endosperm. Furthermore, *GH3.5* expression changes dramatically after fertilization. Instead of being restricted to the embryo sac, it becomes expressed in the apical end of the funiculus (**Figure 4H**). Together this suggests that fertilization triggers a switch or change in the flux of the auxin transport, so that the export from the ovule through the funiculus is repressed, while the spreading of auxin around the differentiating embryo sac is stimulated. Simultaneously, auxin inactivation is repressed in the embryo sac, while it is induced in the upper part of the funiculus, presumably fine-tuning the auxin levels required for embryo and seed development.

## Discussion

**FIGURE 5:**
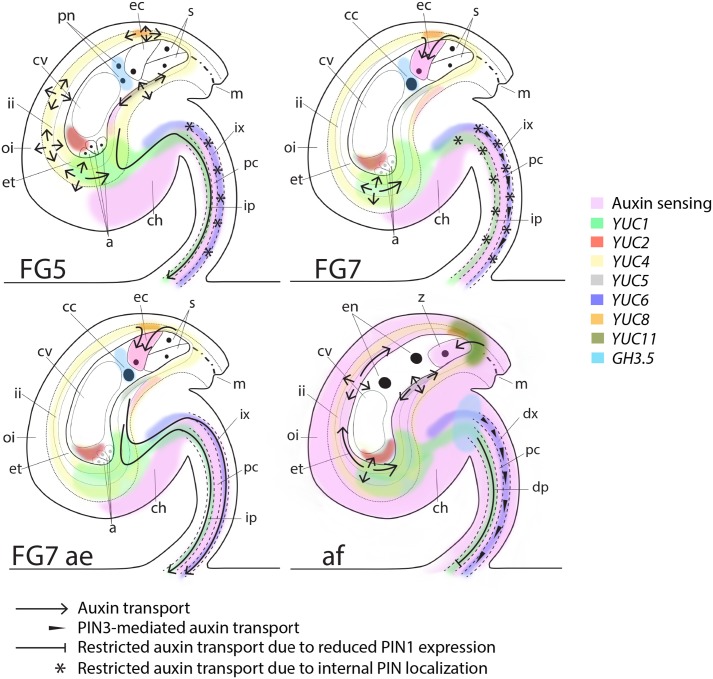
Schematic map of auxin sensing, biosynthesis, conjugation and transport at pre-anthesis, anthesis, after emasculation and after fertilization. At the pre-anthesis stage (FG5), auxin biosynthesis, as observed by *YUC* expression, occurs in the inner integuments, the female gametophyte and in the funicular provasculature (pc; procambium, ip; immature phloem and ix; immature xylem). However, auxin sensing is low in the integuments and in the female gametophyte, presumably due to PGP-mediated auxin transport around the embryo sac (arrows; Lituiev et al., 2013), GH3.5-mediated auxin conjugation in the female gametophyte and PIN-mediated auxin efflux through the immature phloem of the funiculus (arrow). At this stage PIN1, PIN3 and PIN6 are all internally localized in the immature xylem (stars), presumably preventing basal auxin transport in this cell file. At anthesis (FG7), auxin sensing remains low in the integuments and the female gametophyte, but the egg cell starts sensing auxin, which presumably is delivered by AUX1 (Panoli et al., 2015) from the surrounding tissues (arrows). Auxin transport through the immature phloem in the funiculus is repressed by internalization of PIN1 (stars), while basal transport is slightly induced in the immature xylem by increased polarization of PIN3 (arrowheads). If the flowers are emasculated before anthesis (FG7 ae), polar auxin transport through the funicular provasculature is stimulated by basal polarization of PIN1 and PIN6 (arrows), while auxin biosynthesis and auxin sensing in ovules remain unchanged compare to in ovules of non-emasculated flowers. Shortly after fertilization (af), auxin sensing increases drastically throughout the sporophytic tissues of the ovule. Auxin biosynthesis increases in the micropylar end of the integuments through *YUC5* and *YUC11* activation, while GH3.5-mediated auxin conjugation decreases in the endosperm and increases in the apical part of the funiculus. Auxin transport in the differentiating phloem (dp) in the funiculus is restricted through reduced PIN1 expression levels (blocked arrow) and in the differentiating xylem (dx) through reduced PIN1 and PIN6 expression levels. PIN3, however, remains expressed and basally localized in the dx, presumably retaining a certain level of auxin transport in this cell file (arrowheads). In addition, PIN3-mediated auxin transport is induced in the integuments (arrows), presumably to facilitate the spreading of auxin throughout the sporophytic tissues. PIN3 is also expressed and basally localized in cell file 4 of the funiculus throughout all analyzed stages. This suggests that PIN3-mediated auxin transport in the cell file between the differentiating phloem and the differentiating xylem is not important for the changes in ovule development from before anthesis to after fertilization and is therefore not indicated in the model.

The transitions between ovule and seed as well as between gynoecium and fruit are vital for the survival of a plant species. A tight communication between different female reproductive tissues as well as between female and male reproductive organs is essential to allow successful fertilization and subsequent development and growth of embryos, seed and fruit. Although, auxin has been implicated in the coordination of these transition events in different species (Dorcey et al., 2009; Fuentes and Vivian-Smith, 2009; Figueiredo et al., 2016; Goldental-Cohen et al., 2017), the spatiotemporal details, as well as the identity of the main framework for regulatory processes have largely remained elusive. Here we present a first map of auxin biosynthesis, transport, conjugation and sensing shortly before as well as after fertilization in the model plant Arabidopsis (**Figure 5**). Arabidopsis is the principal plant species in which this high resolution spatiotemporal analysis is currently feasible. However, even though the Arabidopsis model is an autogamic species, this map can be used as a foundation for further studies in both other self-fertilizing plants and in allogamous species. The map shows that auxin transport, auxin conjugation and to some extent auxin biosynthesis is highly dynamic in ovules around fertilization. We suggest that these components together regulate the auxin levels important for the developmental decisions in pre-anthesis, anthesis and post-fertilization ovules.

### Auxin Appears to Accumulate in Sporophytic Tissues of the Ovule Already at Anthesis

Whereas the expression of several auxin biosynthesis genes is persistently high in ovules from pre-anthesis to post-fertilization stages, auxin distribution as detected by the *R2D2* sensor is dynamic during the same stages. While DII depletion is restricted to specific tissues of the funiculus, and to some extent to the chalazal domain and the endothelium before fertilization (**Figures 1B,3C**), it becomes highly active in all sporophytic tissues after fertilization (**Figure 4B**), suggesting significant turnover of Aux/IAA proteins throughout the ovule (**Figure 5**).

Both the *R2D2* auxin sensor system and the extensively used *DR5* auxin response reporter are dependent on functional TIR1/AFB proteins (Brunoud et al., 2012). These proteins are subjected to significant post-transcriptional regulation (Parry et al., 2009), and the abundance of both specific members of the TIR1/AFB family the Aux/IAAs can be modulated by the microRNA miR393 (Windels et al., 2014). Furthermore, TIR1/AFB S-nitrosylation enhances TIR1-Aux/IAA interaction (Terrile et al., 2012), and nitric oxide signaling may occur during the time of fertilization (Domingos et al., 2015), potentially influencing the ability of both *R2D2* and *DR5* to sense and report auxin. However, both exogenous auxin application as well as transient inhibition of PAT in non-fertilized ovules by 1-N-1-naphthylphthalamic acid (NPA) treatment results in elevated auxin responses in the integuments and the funiculus (Dorcey et al., 2009; Fuentes and Vivian-Smith, 2009). This indicates that all components required for sensing auxin in those tissues are present already before fertilization, and that the auxin produced by the *YUC* genes expressed in the integuments is actively exported from this specific tissue before fertilization. In pre-anthesis ovules, the auxin efflux carriers ABC-B/MULTI-DRUG RESISTANT/P-GLYCOPROTEIN1 and 19 (ABCB/PGP1 and 19) are expressed in the integuments (Lituiev et al., 2013), while the PIN proteins are mainly active and basally localized in the chalazal end of the ovule and in the funiculus. As NPA-treatment leads to auxin entrapment in both ABCB/PGP and PIN expressing cells (Petrášek et al., 2006; Larsson et al., 2014), these two sets of proteins most likely mediate auxin export from the ovule before anthesis (**Figure 5**).

At stage FG7, PGP19 is no longer active in the inner integuments (Lituiev et al., 2013) and we show that PIN mediated auxin flow through the funiculus is repressed while the expression of auxin biosynthesis genes throughout the ovule remains stable (**Figure 5**). This suggests that even if auxin is canalized away from ovules prior to anthesis, the flow does not seem to be sufficient to completely counteract the biosynthesis rate at anthesis. Thus, the auxin synthesized in the ovule integuments at anthesis could potentially be compartmentalized or metabolized before fertilization. Taken together, this suggests that the auxin produced in the ovules before fertilization is possibly both stored by conjugation or compartmentalization as well as partly exported out of the ovule, counteracting any accumulation of auxin above a threshold necessary to induce DII depletion or a response as monitored by the *DR5* element (**Figure 5**).

### Anthers Regulate Ovule and Gynoecium Development by Controlling Funicular Auxin Flow

Auxin readout, auxin biosynthesis and PAT in the funiculus before anthesis may reflect several biological functions. First, the cell files in the funiculus that sense auxin and express PIN1, PIN3, and PIN6 differentiate into xylem and phloem after fertilization. This is in accordance with previous studies showing that auxin responsiveness and PIN1 expression precede procambium markers in aerial organs such as leaves and gynoecia (Scarpella et al., 2006; Larsson et al., 2014). Interestingly, the funicular cells destined to become phloem express *YUC1* and polarized PIN1, while the cell file that differentiates into xylem expresses *YUC6* and highly internalized PIN1, PIN3, and PIN6 (**Figure 5**). Together this indicates that different modules of auxin biosynthesis and transport mark specific cell differentiation fates in the vasculature, which to our knowledge has not been suggested for vascular patterning in any other aerial organs before.

At anthesis, when the vascular patterning of the funiculus is starting to become evident, PIN1 becomes more internalized throughout the provasculature. At this stage, gynoecium development has to be retarded so that the anthers of the stamens can contact the stigma and allow successful fertilization. Furthermore, at anthesis, and throughout the receptive period, ovule development enters a quiescent period until fertilization takes place. The repressed PIN1-mediated auxin canalization in ovules at anthesis may contribute to the cessation of pre-fertilization growth as aerial organ outgrowth has been shown to be dependent on interior basipetal PIN1 localization and subsequent auxin canalization through the pre-procambium of developing organs, and loss of this basipetal auxin flow results in terminated organ growth (Furutani et al., 2014). Interestingly, Werner et al. (2011) showed that phloem unloading in ovules ceases at anthesis, suggesting that PIN1 internalization might contribute to, or perhaps be a consequence of, the restricted nutrient allocation to ovules at this specific stage. PIN6 was recently reported to have a dual function, depending on its localization either to the plasma membrane or to the ER. The localization was shown to be developmental stage-dependent, and it was suggested that the internal localization can mediate intracellular compartmentalization of free auxin (Simon et al., 2016). Thus, the internal PIN6 expression at anthesis might be a component mediating compartmentalization of auxin that can be released after fertilization when both PIN1 and PIN6 expression in the funiculus is reduced (**Figure 5**).

Strikingly, all PIN proteins expressed in the funiculus (including PIN6) become targeted to the plasma membranes of the funiculus if the flowers are emasculated. This suggests that the outer floral organs (i.e., the stamens) control the flow of auxin through the funiculus as the flower approaches anthesis. The complete mechanism is difficult to envisage but it is interesting to note that an auxin response maxima occurs in the anthers prior to anthesis (Aloni et al., 2006; Cecchetti et al., 2017). However, whether this auxin response maxima contribute to reducing the auxin transport through the funiculus remains to be investigated.

While emasculation of wild type Arabidopsis flowers does not lead to fruit growth, it has a substantial effect on fruit growth in the parthenocarpic *arf8* mutant, and it has been suggested that inter-organ communication regulates gynoecial development until fertilization has taken place (O’Neill, 1997; Vivian-Smith et al., 2001). Thus, together with our observations that emasculation triggers PIN polarization, this suggests that the outer floral organs likely exerts a restriction on the gynoecium growth at the anthesis stage by controlling auxin flow through the funiculus (**Figure 5**). The signaling process may occur directly on the funiculus from the anther, or it may occur indirectly through other tissues of the ovule. Interestingly, the *aberrant testa shape*/*kanadi4* mutant (*ats1-1*/*kan4-1*), which develop ovules with only three layers of integuments (Léon-Kloosterziel et al., 1994), strongly enhances the parthenocarpic fruit growth of *arf8* mutants, even without emasculation (Vivian-Smith et al., 2001). This suggests that the integuments are crucial for the anther-dependent restriction on fruit growth. To our knowledge there are no reports about homeotic stamenless mutants in Arabidopsis that are parthenocarpic, nor are there specific male sterile mutants which are parthenocarpic. However, the *pistillata* (*pi*) mutant in apple (*Malus domestica*) lacks both petals and stamens, while it produces fruits without fertilization (Tobutt, 1994; Yao et al., 2001). Furthermore, in tomato (*Lycopersicon esculentum*) the male sterile mutants, such as *stamenless* (*sl*) and *pistillate* (*pi*) show occasional parthenocarpy (Gomez et al., 1999; Olimpieri and Mazzucato, 2008), supporting our hypothesis that the male reproductive organs can regulate the co-ordination and growth of the female reproductive organs.

### Fertilization Triggers Expression of Auxin Biosynthesis Genes in the Micropylar End of the Ovule, Adjacent to the Basal Part of the Zygote

Embryo development starts with an asymmetric division of the elongated zygote, resulting in a smaller apical cell and a larger basal cell. Already from the first zygotic division on, auxin is transported from the basal cell to the apical cell in a PIN7-dependent manner (Friml et al., 2003).

However, both the origin of the auxin transported to the apical cell and the role of auxin for zygote elongation has remained elusive, although the presence of maternally derived positional signals seems feasible (Zhang and Laux, 2011). Our data show for the first time that the egg cell detects auxin already before fertilization. The auxin influx carrier *AUXIN1* (*AUX1*) accumulates in the micropylar end of the female gametophyte, specifically around the egg cell and the synergids from stage FG6, and auxin has been shown to regulate the specification of these cells (Panoli et al., 2015). Thus, the auxin sensing in the egg cell from this stage might result from auxin influx from both the surrounding integuments and from the central cell (**Figure 5**). Shortly after fertilization, both *YUC11* and *YUC5* are specifically induced in the micropylar end of the ovule, adjacent to the basal end of the zygote (**Figures 4C′,D′**). Furthermore, *YUC8* is expressed in the same area both before and after fertilization (Supplementary Figures 4P–R). This suggests that these three auxin biosynthesis genes are potentially responsible for producing the auxin that is required for embryo polarization after the first division of the zygote.

### Changes in PAT, Auxin Conjugation and Auxin Biosynthesis May Contribute to Rapid DII Depletion after Fertilization

It has previously been shown that significant auxin responses are induced in ovules early after fertilization, and these responses have been suggested to stimulate a cascade of events that leads to both seed coat development and fruit growth (Dorcey et al., 2009; Fuentes and Vivian-Smith, 2009; Figueiredo et al., 2016). Here we could show that after fertilization, PIN1 and PIN6 expression in the funiculus decreases rapidly, while PIN3 expression is induced in the endothelium. At the same time, auxin biosynthesis is induced in the micropylar end and the expression of the auxin conjugating enzyme GH3.5 is repressed in the endosperm and induced in the upper part of the funiculus. Figueiredo et al. (2016) showed that auxin production in the central cell of the female gametophyte is sufficient to drive seed coat development and parthenocarpic fruit growth. Since we can observe expression of auxin biosynthesis genes (*YUC2* and *YUC5*) in the central cell already before fertilization, the putative reduction in auxin conjugation might contribute to a pool of auxin that is required for seed coat development and fruit growth. Furthermore, these activities, together with a putatively reduced compartmentalization of auxin in the integuments, might contribute to the elevated auxin sensing as observed in the sporophytic tissues of the ovule only hours after fertilization (**Figure 5**).

Taken together our data provide a detailed foundation for future studies on auxin-regulated developmental transitions, as well as on ovule, seed and fruit growth.

## Author Contributions

EL designed the experiments and discussed these with AV-S, RO, and ES. EL performed the experiments, analyzed the data, and all authors discussed the results. EL wrote the manuscript. AV-S, RO, and ES commented on the manuscript.

## Conflict of Interest Statement

The authors declare that the research was conducted in the absence of any commercial or financial relationships that could be construed as a potential conflict of interest.
