# Supplementary figures and images for: Auxin Homeostasis in Arabidopsis Ovules Is Anther-Dependent at Maturation and Changes Dynamically upon Fertilization
Source: Front Plant Sci. 2017 Oct 10;8:1735. doi: 10.3389/fpls.2017.01735 (PMC5641375; doi:10.3389/fpls.2017.01735)

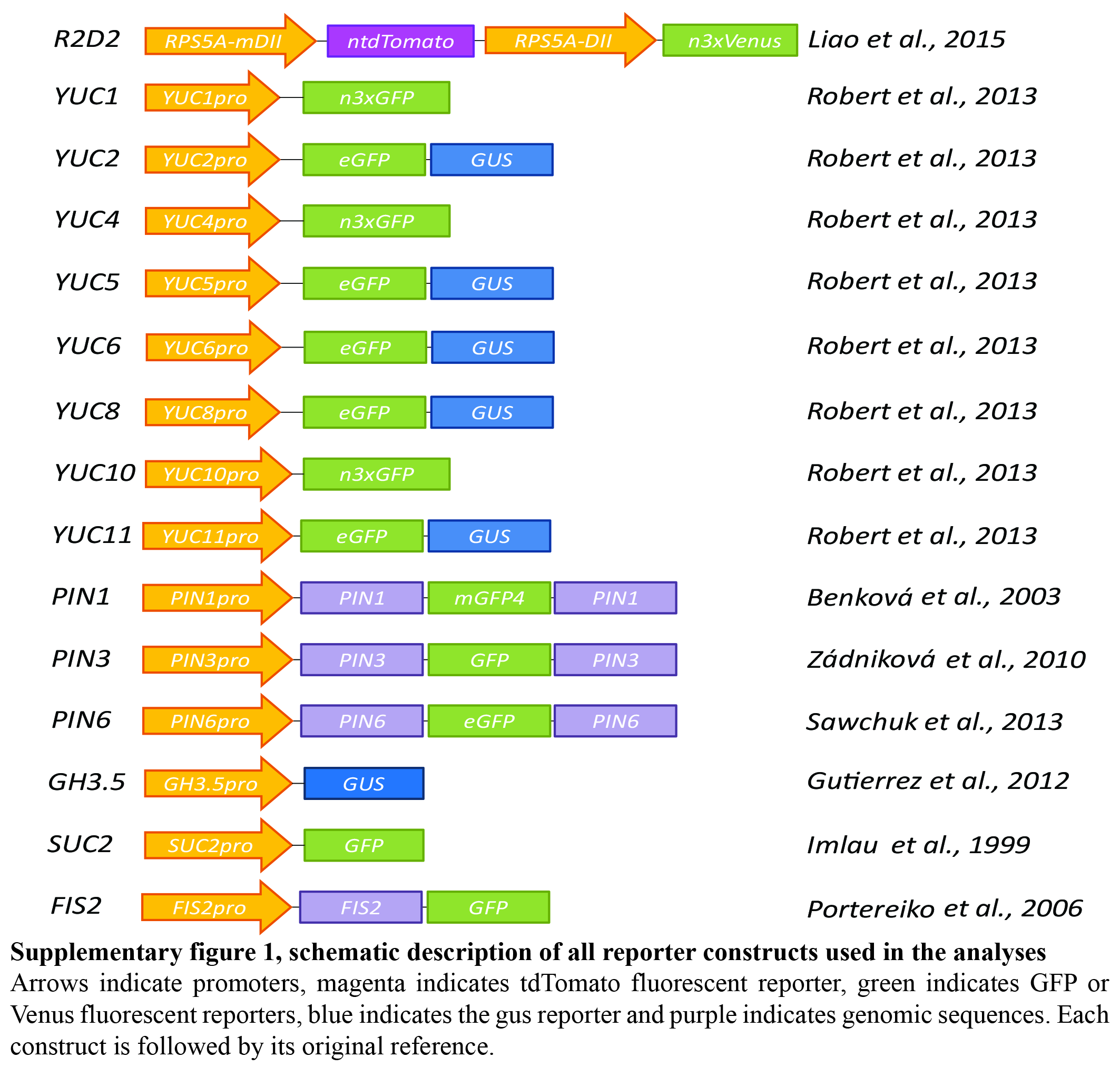

Supplement: Supplementary file 1 [file Image_1.tif]

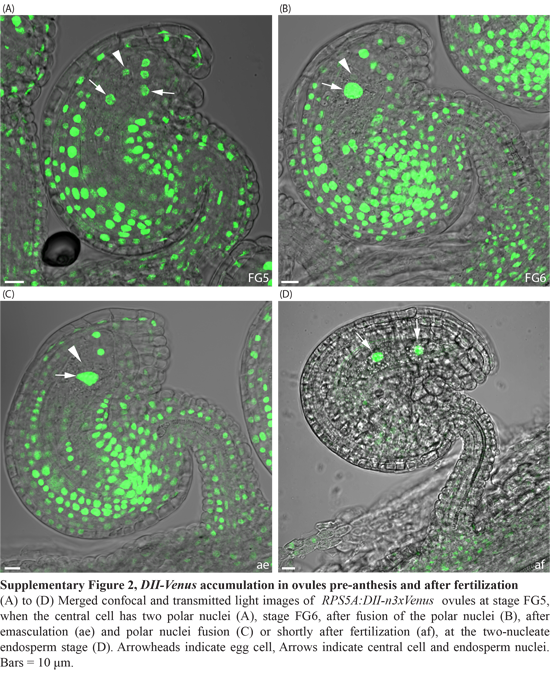

Supplement: Supplementary file 2 [file Image_2.tif]

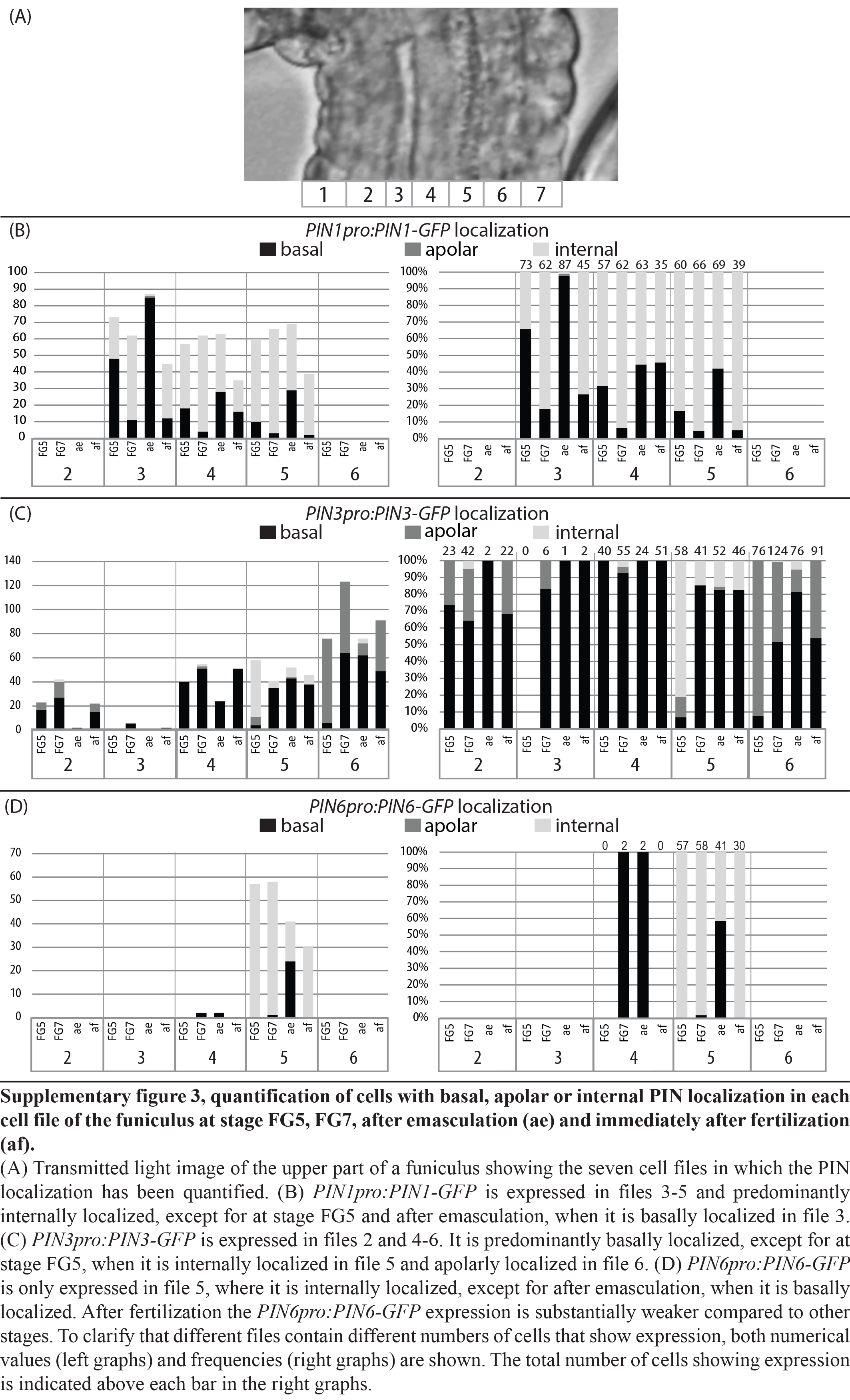

Supplement: Supplementary file 3 [file Image_3.tif]

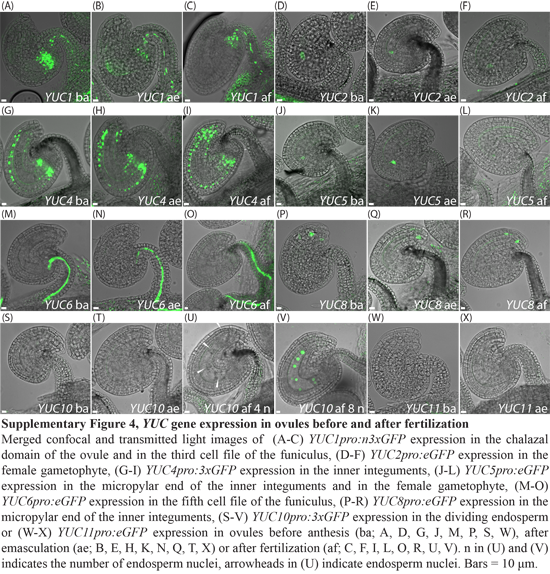

Supplement: Supplementary file 4 [file Image_4.tif]

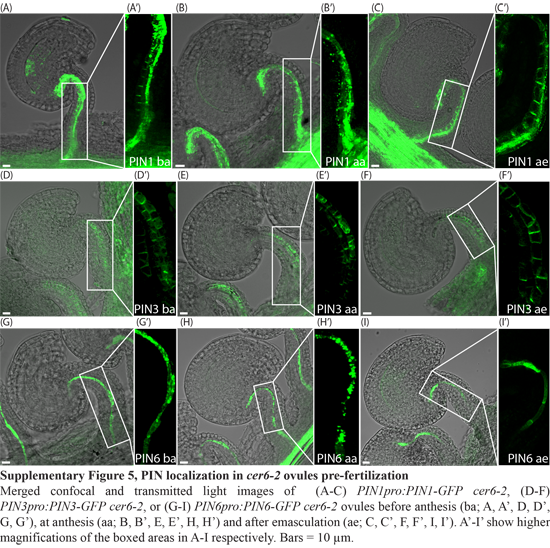

Supplement: Supplementary file 5 [file Image_5.tif]
